# The usefulness and effectiveness of game-based learning when revising and preparing for written exams in nursing education: A feasibility study

**DOI:** 10.1371/journal.pdig.0001043

**Published:** 2025-10-24

**Authors:** Nuno Tavares, Nikki Jarrett

**Affiliations:** 1 School of Dental, Health and Care Professions, University of Portsmouth, Portsmouth, United Kingdom; 2 NIHR ARC Wessex, University of Southampton, Southampton, United Kingdom; Yonsei University College of Medicine, KOREA, REPUBLIC OF

## Abstract

Studying for final exams is often regarded as difficult for nursing students, therefore, activities using game-based learning methods may increase student satisfaction. Therefore, this study aimed to understand the feasibility of a game-based learning activity on nursing students’ learning and revision processes. A one-group pre and post-questionnaire design was undertaken to evaluate the effectiveness of a game-based learning activity. All nursing students found the game-based learning activity valuable when preparing for written exams. The learning activity increased the levels of knowledge retention and the final grades. Although two students found the activity somewhat distracting, most students believed that game-based learning should be embedded into the nursing curriculum. The game-based learning activity was well-accepted when revising for written exams in nursing. However, research at a larger scale is required to confirm the effectiveness of the activity on students’ knowledge, grades and long-term retention.

## Author Summary

Nursing students often find preparing for written exams difficult and stressful. Therefore, we explored if using a game elements in learning activities could make revision more engaging and effective in nursing undergraduate students. Second-year nursing students were invited to take part in a competitive and engaging quiz delivered through Kahoot!, which included questions similar to those in their upcoming exam. The quiz focused on long-term conditions and knowledge was assessed before and after the game-based activity. Student’s views and experiences during the activity were also collected. The results showed that students enjoyed the session, found it useful for identifying areas needing further revision, and valued the opportunity to discuss questions and answers with lecturers. Most participants felt the activity improved their confidence and supported exam preparation, though a few reported that the competitive and time-limited format was distracting. Overall, the study suggests that game-based learning may help students revise in a more interactive and enjoyable way, though larger studies are needed to confirm these findings.

## Introduction

Revising, the process of studying previously learnt knowledge in preparation for an exam [1], has always been regarded by students as a difficult, dull and labour-intensive task [2]. The negative connotation associated with this lonely task often leads to frustration, poor engagement and poorer academic performance in Higher Education [2]. Although preparing for written examinations is a common activity in Higher education, it is particularly complex for healthcare and nursing students, since students need to manage high workloads and acquire large volumes of knowledge in a short period of time [3]. As a consequence, nursing students need to memorise and understand complex and large amounts of information about physiological and pathological processes of the human body that require longer and repeated periods of studying [3,4]. In order to be successful, nursing education requires the active participation and engagement of students to improve overall student attainment [5,6].

Traditional methods of knowledge acquisition have been phased out and new and more engaging and active methods of learning have made their way into higher education [7]. Prolonged periods of studying in the library for long periods of time have been replaced with short learning sessions enabled by digital technologies in the comfort of students’ homes [8]. This has led to changes in the way learning has been taking place and was hastened by the SARS-COV-2 pandemic, where students have grown accustomed to learning from home [8,9].

One way to increase student engagement with their studies is by using game-based learning, which is defined as the use of game-design elements (e.g., competition, leader boards and prizes) to enhance academic performance and learning [10,11]. This approach increases participation and is more attractive to students [5]. Although game elements are used to enhance learning, education remains the primary goal of this approach [11]. Healthcare simulation can make use of some game elements, however, it is not viewed as game-based learning [11].

Game-based learning has been proven to be more effective than traditional teaching methods in improving knowledge retention, motivation and meaningful learning, encouraging critical thinking, decision-making skills and academic performance [12]. Despite this, previous research has highlighted some limitations to game-based learning. The long-term and repeated use of game-based learning is one limitation [13,14]. A recent study suggested that student interest and motivation were lost over time as the same game elements were used throughout the academic year [15]. Another limitation relates to student perceptions of game-based learning. Although students saw game-based learning as a suitable and valuable tool in the classroom, some students questioned the usefulness of this approach when revising and preparing for final exams [16]. Students seemed to associate the use of game elements in learning with recreational games, which made learning fun but removed the seriousness of revising when preparing for exams [4,12].

This research study attempts to address and explore some of the limitations of game-based learning highlighted above. The study aims to explore the usefulness and impact of game-based learning when revising and preparing for academic written examinations in the nursing curriculum. Although game-based learning is well known in the nursing literature, its application in revising for final exams has never been explored in nursing education. A systematic literature review suggested that previous game-based learning research focused on developing or consolidating nursing skills but did not explore the use of this approach when revising for final written exams [4]. 

## Methods

### Ethics statement

Ethical approval was provided by the Science and Health Faculty Ethics Committee at the University of Portsmouth in March 2022. Written consent was sought from all participants at least 24 hours before the start of the study.

### Sample

A total of 149 second-year nursing students completing the Adult Nursing Programme (Baccalaureate Nursing Program) and the module entitled “Nursing People with Long-term Conditions” were invited to participate. The module was part of the curriculum and delivered in the second year of the programme.

### Research design

The feasibility study used a one-group pre and post-questionnaire design where a non-randomised intervention was implemented. Pre- and post-knowledge tests and questionnaires were conducted to understand the usefulness and impact of GBL on the revision process (please refer to S1 File to find the pre and post knowledge test).

Participants were invited to attend an activity (quiz), which contained 30 multiple-choice and true-and-false questions delivered using Kahoot!. The intervention was conducted at a time when students were revising for their summative written exam (April 2022). The intervention was not part of normal teaching.

The quiz focused on the content taught in the aforementioned module and were randomly generated from a bank of questions that was used to develop the summative exam (please refer to S2 File to find the quiz). Therefore, there were no real differences between the complexity and content of the questions in the game-based quiz and the final written exam.

### Intervention

Participants were asked to collaboratively attempt to discuss and answer each question within the time limit available (1 min per question). The facilitators (lecturers on the module, including NT) shouted out the questions and students were awarded points for their answers. Kahoot! was used to generate competitiveness, by awarding points to correct answers and rewarding quick response times.

At the end of each question, students were made aware of their scores on a leaderboard and discussed the question, the answers provided, and the correct answer. Each question was unpicked and its content and format discussed to improve student interpretation and overall clarity. The discussion used active learning methods and was thought to enhance knowledge retention, consolidation, and conceptualization. The group of participants with the highest score won the competition and received prizes, including Amazon vouchers, chocolates, or coffee vouchers.

### Recruitment strategy

Students were invited to participate in the research study via email. A lecturer on the module and outside of the research team shared information about the study with the nursing students. Students willing to participate were asked to contact the principal investigator (NT). Further information about the study was provided to all potential participants and any questions answered.

### Data collection

At the start and end of the activity, students completed a knowledge test to understand the impact of the intervention on knowledge retention. In addition, students were asked to complete a learning experience questionnaire exploring gameful experiences (GAMEX) [17] and their perceptions of the usefulness of the session when revising for summative (please refer to S3 File to find the learning experience questionnaire). The GAMEX scale was validated with a Cronbach α value of 0.855 (with values > 0.7 considered acceptable) and a correlation level of for test-retest reliability of 0.89 (p < 0.05) [17].

The last element of data collection included collecting participants’ performance in their final exams. Participants’ grades on the final written exam were compared with the average grade for the module, which included the grades of all students completing the aforementioned module. All students consented to share the average final grade of the module to the research study.

### Data analysis

The data collected encompassed quantitative and qualitative data. Quantitative data were analysed using descriptive and inferential statistics (such as mean, standard deviation) and qualitative data collected through open questions were analysed using principles of thematic analysis. Due to the limited amount of qualitative data provided by the participants, it was not possible to complete all steps of thematic analysis, particularly the construction of inductive themes. Instead, qualitative data was analysed and synthesised to complement and provide context to the quantitative data.

### Findings

A total of 9 participants out of 149 students accepted to take part in the research study. All participants completed all elements of the research study. Therefore, no data was missing from the data collection tools. Please refer to **Table 1** to find information about the participants’ characteristics.

**Table 1:** Participant characteristics.

Number of participants (percentage of students recruited)	9 (6%)
Gender	Female - 8 Male - 1
Average Age (SD)	36 (7.95)

### Gameful experience

**Table 2** presents the average scores on the GAMEX scale contained in the Learning Experience Questionnaire, which measured participants’ gameful experience in six domains.

**Table 2:** Average scores of the GAMEX scale contained in the Learning Experience Questionnaire.

Items	Mean	Standard Deviation
Enjoyment Section	4.93	0.18
Item 1 - Playing the game was fun	5	0
Item 2 - I liked playing the game	5	0
Item 3 - I enjoyed playing the game very much	5	0
Item 4 - My game experience was pleasurable	5	0
Item 5 - I think playing the game is very entertaining	4.56	1.33
Item 6 - I would play this game for its own sake, not only when being asked to.	5	0
Absorption Section	3.54	0.41
Item 7 - Playing the game made me forget where I am.	3.12	1.32
Item 8 - I forgot about my immediate surroundings while I played the game.	3.11	1.45
Item 9 - After playing the game, I felt like coming back to the “real world” after a journey.	3.56	1.24
Item 10 - Playing the game “got me away from it all”	3.56	1.24
Item 11 - While playing the game I was completely oblivious to everything around me.	3.67	1.41
Item 12 - While playing the game I lost track of time.	4.22	0.83
Creative Thinking Section	4.14	0.17
Item 13 - Playing the game sparked my imagination.	4.22	0.83
Item 14 - While playing the game I felt creative.	3.89	0.93
Item 15 - While playing the game I felt that I could explore things.	4.22	0.97
Item 16 - While playing the game I felt adventurous.	4.22	0.83
Activation Section	3.69	1.00
Item 17 - While playing the game I felt activated.	4.44	0.88
Item 18 - While playing the game I felt jittery.	2.89	1.62
Item 19 - While playing the game I felt frenzied.	2.78	1.56
Item 20 - While playing the game I felt excited.	4.67	0.71
Absence of Negative Effect Section	1.37	0.39
Item 21 - While playing the game I felt upset.	1.33	1.00
Item 22 - While playing the game I felt hostile.	1.00	0.00
Item 23 - While playing the game I felt frustrated.	1.78	1.09
Dominance Section	3.33	0.67
Item 24 - While playing the game I felt dominant/I had the feeling of being in charge.	2.89	1.83
Item 25 - While playing the game I felt influential.	3.67	1.41
Item 26 - While playing the game I felt autonomous.	2.67	1.50
Item 27 - While playing the game I felt confident.	4.11	1.05

**Key:** Students rated each question using the following Lickert scale:

- Strongly Agree – 5.

- Agree – 4.

- Neutral – 3.

- Disagree – 2.

- Strongly Disagree – 1.

- Don’t know/Not applicable - DN/NA.

The findings from the GAMEX scale suggest that students found the game-based activity enjoyable and with a creative thinking potential. The activity seemed to provide moderate absorption, dominance and activation. Lastly, the participants did not report any adverse effects of the activity on themselves, although a few felt somewhat frustrated with their performance on the quiz. The scores across and within participants seemed randomly distributed and there was no correlation between their answers and their individual characteristics.

Participants commented briefly on the answers provided to the scale, which helped explain the scores provided. Most participants commented on how much they enjoyed the activity and how it suited their learning style.

*“I find learning more fun with games which enables me to retain information more. I also learn best from my mistakes so will go and research what I do not know or expand my knowledge.”* (Participant 4)

However, a few participants felt that the time limit and the competitive element of the game-based activity led them to pay less attention to the questions and seemed somewhat distracting, so some students answered questions using their instinct rather than comprehensive thinking.

*“The quiz felt playful yet intense, because of the time limit for each question I found that I was listening to my instinct more instead of overthinking. The questions also opened up opportunities to ask further relevant questions.”* (Participant 5)

### Usefulness and appropriateness of the game-based learning

**Table 3** displays participants’ views on the usefulness and appropriateness of the game-based activity when revising and preparing for the summative exam. These questions comprised the second part of the Learning Experience Questionnaire.

**Table 3:** Participants’ views on the appropriateness and usefulness of the intervention when preparing for their final exam.

Items	Mean	Standard Deviation
Q1. I/Our group did well in the activity.	4.22	1.30
Q2. I have learnt from the activity.	4.89	0.33
Q3. The activity was a useful learning opportunity.	4.89	0.33
Q4. I **don’t think** these activities are helpful when preparing for exams.	2.44	1.94
Q5. The activity provided a useful opportunity to revise and prepare for the exam.	4.89	0.33
Q6. Which aspects of the activity did you enjoy the most (grade each element)?		
Interaction with fellow students	All participants replied - Enjoyed the most	
The game itself	All participants replied - Enjoyed the most	
Type of questions	All participants replied - Enjoyed the most	
Content covered in the session	All participants replied - Enjoyed the most	
Discussion at the end	9 participants replied - Enjoyed the most 1 participant replied - Somewhat enjoyed it	
Q7. The activity has allowed me to reflect on how to structure and plan my revision.	4.50	0.71
Q8. The activity allowed me to understand which areas **require further revision**.	5.00	0
Q9. The activity was useful to understand which areas **do not require further revision**.	3.78	1.72
Q10. As a result of this activity, I feel more confident about revising and preparing for the exam.	4.78	0.44
Q11. As a consequence of this activity, I will spend **more time** revising for the exam.	4.89	0.33
Q12. As a result of this activity, I will spend **less time** revising for the exam.	1.56	1.33
Q13. The activity suited my learning style.	4.89	0.33
Q14. I would have preferred more interaction in the session.	2.11	1.36
Q15. I would have preferred a quieter activity.	1.44	1.01
Q16. I would like more of these sessions in the course.	4.89	0.33
Q17. These activities are only useful if learning new content.	1.56	1.33

**Key:** Students rated each question using the following Lickert scale:

- Strongly Agree – 5.

- Agree – 4.

- Neutral – 3.

- Disagree – 2.

- Strongly Disagree – 1.

- Don’t know/Not applicable - DN/NA.

The participants found the activity helpful to understand which areas required further revision. In addition, most participants believed they will spend more time revising for the final exam as a result of completing the activity.

*“The activity (shed) light on things and topics that l need more revision.”* (Participant 8)

Some participants believed that the activity allowed them to develop their confidence levels when preparing for the final exam, as it provided them with tools to better understand its requirements and what to expect.

*“I think these sessions are very useful and help in gaining confidence for (the) exam.”* (Participant 3)

The structure and content of the activity seemed to have been well accepted by the students, who believed that the amount of interaction and discussion was just right.

*“The learning (quiz) and explanation (discussion) parts of the session (were the most helpful) because it helps us understand the questions more.”* (Participant 7)

Lastly, participants shared a desire to embed further game-based sessions into the curriculum, in order to conceptualise the content learnt in each module. The activity was found useful when consolidating knowledge and revising learnt content.

*“It would be great to do them (game-based activities) after each topic to see how much information we retained and where we could do some further reading.”* (Participant 4)

### Impact on knowledge retention

Participants’ knowledge was tested before and after the intervention using the same questions in the pre and post-knowledge test. The results of the pre and post-knowledge test can be found in **Table 4**.

**Table 4:** Results of the pre and post-knowledge tests.

Participants	Pre-knowledge test	Post-knowledge test
Participant 1	75/100	70/100
Participant 2	40/100	55/100
Participant 3	65/100	65/100
Participant 4	45/100	60/100
Participant 5	70/100	90/100
Participant 6	80/100	80/100
Participant 7	55/100	65/100
Participant 8	55/100	55/100
Participant 9	40/100	40/100
**Average (SD)**	**58/100 (16)**	**64/100 (15)**

**Fig 1:**
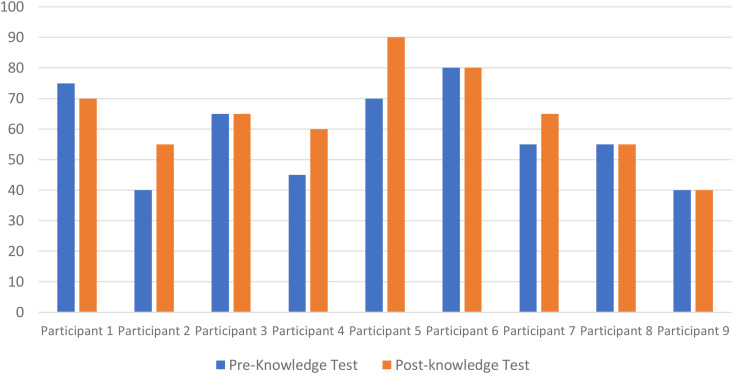
Comparison between pre and post-knowledge test scores.

Overall, the results show a general increase in students’ knowledge level after completing the game-based activity (please refer to Fig 1 for a more detailed comparison between pre and post scores). The results show that only one student performed worse in the post-knowledge when compared to the pre-knowledge test and that students either had similar results (4 participants) or were able to perform better in the post-knowledge test (4 participants). Interestingly, while some participants were able to provide correct answers to the questions in the post-knowledge that they had answered incorrectly in the pre-knowledge test; other students answered incorrectly questions in the post-knowledge that they had answered correctly in the pre-knowledge test. There was no apparent correlation between the changes in their answers and their overall result in the knowledge test.

When it comes to long-term retention and the impact of the activity on student results on the final exam, the cohort’s average mark for the final exam was 61.0, while the average of the students that decided to partake in the study was 74.4. The result suggested that the activity had a positive impact on students’ preparation for their final exam. It was unclear, however, which part of the activity had the largest impact on the results of the final exam - the quiz, discussion, revision or other factors. As some students suggested, the activity may have pinpointed areas that required further revising and students may have acted upon this extra information.

## Discussion

The research study aimed at understanding the feasibility of a game-based learning activity - a quiz - when preparing for written exams. The results suggest that students enjoyed and found the activity useful when preparing for these exams. The activity allowed students to revise and consolidate knowledge, whilst highlighting areas that required further study. The results also suggest a positive impact on knowledge retention and the grade obtained in the final exams. The activity was well-accepted and students requested the addition of more of these sessions into the curriculum. Despite the encouraging results, a small proportion of students found the activity distracting since they felt they were not able to concentrate fully on the task.

The results from this study suggest that students enjoyed revising for their final exam using a game-based learning activity. Previous studies have also highlighted the enjoyment that students experience when taking part in game-based learning activities. As an example, previous research suggested that students found game-based learning activities enjoyable, especially when compared to non-game-based learning activities [6,16,18]. Similar to the findings of this study, Castro et al. and Kubin have found that students would like and benefit from further game-based learning activities in the nursing curriculum [19,20].

Previous nursing studies have focused on using game-based learning to develop clinical skills and acquire new knowledge [16,21]. Whereas, this study has focused on exploring the use of game-based learning when revising and preparing for written nursing exams. Contrary to students’ beliefs in a previous research study [16], game-based learning was well-accepted by nursing students as an enjoyable and useful learning method when preparing and revising for written exams. Although some students found the activity somewhat distracting, most students found it useful to understand the final exam, discuss each question and have fun whilst revising.

The findings from the study suggest that a few students found the time-limit element of the game-based learning activity distracting and difficult to manage. Time-limited questions are a common element of game-based learning, which has been used in previous research to increase the levels of competitiveness and absorption in the activity [19]. Despite their common use, other research have identified similar issues regarding their use in educational activities [18]. The distracting effect of time-limit questions may impact students’ ability to retain and conceptualise information. This game element seems to increase the levels of anxiety, which in turn leads to poor levels of concentration, deep thinking and troubleshooting [16]. This may be particularly problematic for interventions aimed at improving knowledge retention in students, such as this one. The limited improvement observed in post-intervention levels of knowledge retention may have been a result of the negative impact of the overall effect of the game-based learning activity. Further research should explore whether removing game elements that increase levels of distraction and anxiety can increase knowledge retention and conceptualisation.

## Limitations

A limitation of the study is the small sample size. Although a total of 149 students were signposted to the study, only a small proportion consented and participated. Several reasons related to the timing of the session seemed to have led to the small sample size. The activity occurred at a time when students were busy learning new clinical skills and preparing and completing assessments for various modules. In addition, this session was scheduled for the Easter break and many students travelled back home. Lastly, this session occurred at a time when the COVID-19 pandemic had a peak of new cases in the UK. Alternative dates were sought, however, due to other teaching activities, it was not possible to postpone the session.

## Conclusion

The game-based learning activity was well-accepted, enjoyable and helpful when preparing and revising for written exams according to nursing students. The activity had a small, but positive effect on knowledge retention and consolidation, and seemed to have a positive impact on students’ grades on the summative exam. Longitudinal studies across multiple sites are required to understand the impact of the activity on students’ gameful experience, knowledge, grades and long-term retention.

## Supporting information

S1 File Pre and post-knowledge test. (DOCX)

S2 File 30-question quiz. (DOCX)

S3 File Learning experience questionnaire. (DOCX)

S1 Data Anonymised Research Data (XLSX)
