# Supplementary material for: The usefulness and effectiveness of game-based learning when revising and preparing for written exams in nursing education: A feasibility study
Source: PLOS Digit Health. 2025 Oct 24;4(10):e0001043. doi: 10.1371/journal.pdig.0001043 (PMC12551832; doi:10.1371/journal.pdig.0001043)
Supplement: S1 File — (DOCX) [file pdig.0001043.s001.docx]

### S1 Questionnaire - Pre and post-knowledge test

The knowledge test was administered before and after the quiz. Students were asked to complete the test individually for approximately 20 minutes.

Please note that the questions highlighted in yellow are the correct answers.

**TIA is typically defined as a transient inflammatory attack.**

1. True
2. False

**Which of the following aspects isn’t a health risk associated with loneliness?** (select one)

1. Increased mortality risk
2. Coronary heart disease
3. Depression
4. Cancer

**Motivators - the belief that the individual is in charge of his own health - can encourage patients to adopt self-management behaviours.**

1. True
2. False

**John is 73 years old and has a past medical history of hypertension, type 2 diabetes, chronic kidney disease and myocardial infarction. Which pharmacokinetic stage is affected by John’s past medical history?** (select one)

1. Absorption
2. Distribution
3. Metabolization
4. Elimination

**De-prescribing is the process of withdrawing prescribing competencies from healthcare professionals that failed to appropriately manage polypharmacy**

1. True
2. False

**Coronary artery disease can lead to:** (select all that apply)

1. Ischaemia
2. Infarction
3. Cancer
4. Necrosis
5. Coronary infection

**John has been admitted to ED with chest pain, which radiates to his left arm. Which elements should form part of your initial assessment?** (select all that apply)

1. Neurological observations
2. A-E assessment
3. 12 lead ECG
4. Pressure ulcer assessment
5. Falls risk assessment

**Dementia is a syndrome – usually of a chronic or progressive nature – in which there is deterioration in cognitive function beyond what might be expected from normal ageing.**

1. True
2. False

**Dementia results from a variety of diseases and injuries that primarily or secondarily affect the brain, such as Alzheimer's disease or stroke.**

1. True
2. False

**Which of the following aren’t common symptoms of Dementia?** (select all that apply)

1. Memory loss
2. Thinking difficulties
3. Slurred speech
4. Gradual loss of skills
5. Vision impairment

**Which of the following isn’t a long-term neurological condition?** (select one)

1. Stroke
2. COPD
3. Parkison’s disease
4. Motor neurone disease

**Which of the following are common preparations for insulin?** (select all that apply)

1. Prandial bolus
2. Glandular derivate
3. Basal
4. Biphasic
5. Glucagon-like insulin

**What is the approximate proportion of the diabetes population living with type 2 Diabetes Mellitus?** (select one)

1. 90%
2. 100%
3. 75%
4. 53%

**Which of the following isn’t a type of T2DM management?** (select one)

1. High carbohydrate diet
2. Exercise
3. Oral medication
4. Injectable medication

**Which of the following are common signs/symptoms of frailty?** (select all that apply)

1. Weight loss
2. Increased muscle strength
3. High physical activity levels
4. Reduced gait speed
5. Exhaustion

**Hemorrhagic strokes are commonly treated with thrombolytic therapy.**

1. True
2. False

**Which of the following isn’t a stage of the normal process of swallowing?** (select one)

1. Preparatory stage
2. Oral stage
3. Pharyngeal stage
4. Recoil stage

**Pulmonary rehabilitation can help patients with which of the following elements?** (select all that apply)

1. Education
2. Vaccination
3. Medication optimization
4. Exercise tolerance
5. Breathlessness

**Which of the following are considered signs of increased COPD severity?** (select all that apply)

1. Recurrent chest infections
2. Refractory breathlessness
3. Long-term antibiotics
4. Increased activity levels
5. Long-term oxygen

**An advance decision must relate to a specific treatment or circumstance that patients wish to receive in the future.**

1. True
2. False
