# Supplementary material for: The usefulness and effectiveness of game-based learning when revising and preparing for written exams in nursing education: A feasibility study
Source: PLOS Digit Health. 2025 Oct 24;4(10):e0001043. doi: 10.1371/journal.pdig.0001043 (PMC12551832; doi:10.1371/journal.pdig.0001043)
Supplement: S2 File — (DOCX) [file pdig.0001043.s002.docx]

### S2 Questionnaire - 30-question Quiz

Please note that the questions highlighted in yellow are the correct answers.

**Which elements impact ageing?** (select all that apply)

1. Genetics
2. Age-related diseases
3. Regenerative medicine
4. Cell Biology
5. Demographics

**Which of the following factors are part of self-management?** (select all that apply)

1. Self-efficacy
2. Social Media
3. Emotional aspects
4. Healthcare professionals making decisions for patients
5. Social Networks
6. Disease information and understanding

**One’s belief that they have sufficient willpower to be able to eat less in order to lose weight is considered…** (select one)

1. Motivation
2. Self-efficacy
3. Self-care
4. Empowerment

**Mary is 78 years old and has a past medical history of Dementia, COPD, Hypertension, type 2 diabetes and myocardial infarction. Which of the following factors make Mary at an increased risk of adverse drug reactions:** (select all that apply)

1. Multimorbidity
2. Dementia
3. Supportive family
4. Frailty
5. Obesity

**Which of the following elements are associated with appropriate prescribing?** (select all that apply)

1. Establishing an accurate diagnosis
2. Medicines cause adverse drug reactions
3. Complex medicine regimen
4. Selecting appropriate medicine for the person’s age
5. Titrate doses appropriately

**Robert is a 73-year-old with multiple long-term conditions. How can the nurse increase his risk of polypharmacy?** (select all that apply)

1. Recommend further medicines to treat side effects from John’s medication
2. Explain side effects
3. Monitor new signs and symptoms
4. Work closely with other professionals to optimise therapeutic regimen
5. Let Robert manage his medications independently

**Atherosclerotic plaque can lead to…** (select all that apply)

1. Cerebrovascular accidents in the heart
2. Peripheral vascular disease
3. Acute coronary syndrome
4. Deep vein thrombosis
5. Liver cancer

**What are the main symptoms of myocardial infarction?** (select all that apply)

1. Stabbing pain in the abdomen
2. Tightness and heaviness in the chest or upper abdomen
3. Hemiparesis
4. Radiating pain to the throat, arm or back
5. Productive cough

**Unstable angina can…** (select all that apply)

1. Decrease in frequency and severity
2. Occur recurrently and unpredictably
3. Pain becoming less responsive to GTN spray
4. Pain becoming more responsive to GTN spray

**Which of the following are medicines used to treat heart conditions?** (select all that apply)

1. ACE inhibitors
2. Antiarrhythmic medicines
3. Mucolytics
4. Beta-blockers
5. Calcium channel blockers

**Which of the following are long-term results of myocardial infarction?** (select all that apply)

1. Heart Failure
2. Hypotension
3. Dysrhythmia
4. Cardiac arrest
5. Cardiac regurgitation

**Which of the following are common types of Dementia?** (select all that apply)

1. Huntington’s Dementia
2. Vascular Dementia
3. Alzheimer’s disease
4. Occipital Dementia
5. Parkinson’s disease

**Which of the following are common causes of vascular dementia?** (select all that apply)

1. Reduce blood supply in the brain
2. Hypotension
3. Peripheral vascular disease
4. Acute kidney injury
5. Encephalopathy

**What is a common symptom in patients with Lewy body dementia?** (select one)

1. Visual hallucinations
2. Depression
3. Anxiety
4. Burnout

**In Parkinson’s disease, patients have low levels of adrenaline**.

1. True
2. False

**To which pharmaceutical group does insulin belong to?** (select one)

1. Exogenous insulin preparation
2. Inhibitors of glucose absorption
3. Glucagon-like peptide
4. Insulin secretagogues

**Type 2 diabetes is an autoimmune disease which leads to insulin resistance.**

1. True
2. False

**Which of the following are common signs/symptoms of hypoglycaemia?** (select all that apply)

1. Sweating
2. Shaking
3. Confusion
4. High blood pressure
5. Difficulty speaking

**When are glycemic levels considered to be hyperglycemic?** (select one)

1. 4-6 mmol/L
2. >6 mmol/L
3. >4 mmol/L
4. <4 mmol/L
5. Depends on the individual

**Hyperglycaemic hyperosmolar state is characterised by very low glycemic levels (<2 mmol/L).**

1. True
2. False

**Frail patients are at increased risk of which of the following elements?** (select all that apply)

1. Hospital-acquired infection
2. Cancer
3. Delirium (new confusion)
4. Rapidly become dependent
5. Institutionalisation

**Which of the following are common signs/symptoms of a stroke?** (select all that apply)

1. Hoarse voice
2. Hemiplegia
3. Slurred speech
4. Facial droop
5. Chest pain

**Alteplase assists in the breakdown of fibrin and clot formation and is commonly used in the treatment of hemorrhagic strokes.**

1. True
2. False

**Thrombectomy is the process of mechanical retrieval of a cerebral blood clot.**

1. True
2. False

**Which of the following steps can mitigate the effects of dysphasia?** (select all that apply)

1. Sitting at 45 degrees
2. Avoid distractions
3. Assist the person when required
4. Offer larger chunks of food
5. Modify diet and fluid in line with SLT advice

**Lung function tests are the gold standard for diagnosing COPD**

1. True
2. False

**Inhaled salbutamol can cause which of the following effects?** (select all that apply)

1. Relaxation of the smooth muscle
2. Bronchodilation
3. Bronchoconstriction
4. Bronchospasm
5. Reduced breathlessness

**Inhaled corticosteroids can help with which of the following elements?** (select all that apply)

1. Reduce inflammation in the airways
2. Increase airway responsiveness
3. Reduce the severity of exacerbations
4. Increased hospital admissions
5. Reduce the frequency of exacerbations

**Pulmonary emphysema typically causes chronic cough and mucus hypersecretion.**

1. True
2. False

**Advanced care planning allows people to communicate what’s important to them in case they can’t make a decision for themselves.**

1. True
2. False
