# Supplementary material for: The usefulness and effectiveness of game-based learning when revising and preparing for written exams in nursing education: A feasibility study
Source: PLOS Digit Health. 2025 Oct 24;4(10):e0001043. doi: 10.1371/journal.pdig.0001043 (PMC12551832; doi:10.1371/journal.pdig.0001043)
Supplement: S3 File — (DOCX) [file pdig.0001043.s003.docx]

### S3 Questionnaire - Learning Experience Questionnaire

Section 1 of the questionnaire explored students’ gameful experiences through the use of the Gameful Experience Scale (GAMEX) (Márquez-Hernández, et al. 2019). Section 2 of the questionnaire explored students’ perceptions about the usefulness of game-based learning when revising and preparing for exams.

Before you start

Q1. What is your student number?

- [Open answer box]

Q2. What is your gender?

1. Female 2. Male 3. Other

Q3. What is your age in years?

- [Open answer box]

Section 1 - Gameful Experience

A strongly agree to strongly disagree Lickert scale will be used in the questionnaire, as follows:

- Strongly Agree
- Agree
- Neutral
- Disagree
- Strongly Disagree
- Don’t know/Not applicable

**Enjoyment**

Q1. Playing the activity was fun

Q2. I liked playing the activity.

Q3. I enjoyed playing the activity very much.

Q4. My activity experience was pleasurable.

Q5. I think playing the activity is very entertaining.

Q6. I would play this activity for its own sake, not only when being asked to.

Please use the box below to provide further information about your answers above (optional).

**Absorption**

Q7. Playing the activity made me forget where I am.

Q8. I forgot about my immediate surroundings while I played the activity.

Q9. After playing the activity, I felt like coming back to the “real world” after a journey.

Q10. Playing the activity “got me away from it all”

Q11. While playing the activity I was completely oblivious to everything around me.

Q12. While playing the activity I lost track of time.

Please use the box below to provide further information about your answers above (optional).

**Creative thinking**

Q13. Playing the activity sparked my imagination.

Q14. While playing the activity I felt creative.

Q15. While playing the activity I felt that I could explore things.

Q16. While playing the activity I felt adventurous.

Please use the box below to provide further information about your answers above (optional).

**Activation**

Q17. While playing the activity I felt activated.

Q18. While playing the activity I felt jittery.

Q19. While playing the activity I felt frenzied.

Q20. While playing the activity I felt excited.

Please use the box below to provide further information about your answers above (optional).

**Absence of negative affect**

Q21. While playing the activity I felt upset.

Q22. While playing the activity I felt hostile.

Q23. While playing the activity I felt frustrated.

Please use the box below to provide further information about your answers above (optional).

**Dominance**

Q24. While playing the activity I felt dominant/I had the feeling of being in charge.

Q25. While playing the activity I felt influential.

Q26. While playing the activity I felt autonomous.

Q27. While playing the activity I felt confident.

Please use the box below to provide further information about your answers above (optional).

Section 2 - Usefulness of game-based learning when preparing and revising for exams

A strongly agree to strongly disagree Lickert scale will be used in the questionnaire.

Q1. I/Our group did well in the activity.

Q2. I have learnt from the activity

Q3. The activity was a useful learning opportunity.

Q4. I **don’t think** these activities are helpful when preparing for exams.

Q5. The activity provided a useful opportunity to revise and prepare for the exam.

Q6. Which aspects of the activity did you enjoy the most (grade each element)?

- Interaction with fellow students
- Game
- Type of questions
- Content covered in the session
- Discussion at the end

Q7. The activity has allowed me to reflect on how to structure and plan my revision.

Q6. The activity allowed me to understand which areas **require further revision**.

Q8. The activity was useful to understand which areas **do not require further revision**.

Q9. As a result of this activity, I feel more confident about revising and preparing for the exam.

Q10. As a consequence of this activity, I will spend **more time** revising for the exam.

Q11. As a result of this activity, I will spend **less time** revising for the exam.

Q12. The activity suited my learning style.

Q13. I would have preferred more interaction in the session.

Q14. I would have preferred a quieter activity.

Q15. I would like more of these sessions in the course.

Q16. These activities are only useful if learning new content.

Please use the box below to provide further information about your answers (optional).

Which areas require further improvement? (optional)

Which parts of the session did you like the most or the least? (optional)

If you found the session useful, could you suggest any strategies to increase the number of these sessions in the nursing course? (optional)
